# Outcome selection for tissue-agnostic drug trials for immune-mediated inflammatory diseases: a systematic review of core outcome sets and regulatory guidance

**DOI:** 10.1186/s13063-022-06000-w

**Published:** 2022-01-15

**Authors:** Olalekan Lee Aiyegbusi, Lavinia Ferrante di Ruffano, Ameeta Retzer, Philip N. Newsome, Christopher D. Buckley, Melanie J. Calvert

**Affiliations:** 1 Centre for Patient Reported Outcomes Research, Institute of Applied Health Research, University of Birmingham, B15 2TT, Birmingham, UK; 2 National Institute for Health Research (NIHR) Applied Research Centre West Midlands, and National Institute for Health Research Surgical Reconstruction and Microbiology Research Centre, University of Birmingham, Birmingham, UK; 3 National Institute for Health Research Birmingham Biomedical Research Centre, University of Birmingham, Birmingham, UK; 4 Birmingham Health Partners Centre for Regulatory Science and Innovation, University of Birmingham, Birmingham, UK; 5 Centre for Liver and Gastrointestinal Research, Institute of Immunology and Immunotherapy, University of Birmingham, Birmingham, UK; 6 Liver Unit, University Hospitals Birmingham NHS Foundation Trust, Birmingham, UK; 7 The Kennedy Institute of Rheumatology, University of Oxford, Oxford, UK; 8 Rheumatology Research Group, Institute for Inflammation and Ageing, College of Medical and Dental Sciences, University of Birmingham, Queen Elizabeth Hospital, Birmingham, UK

**Keywords:** Tissue-agnostic clinical trials, Core outcome set, COS, IMIDs, Rheumatoid arthritis, Rheumatology

## Abstract

**Background:**

Tissue-agnostic drug development provides a paradigm shift in precision medicine and requires innovative trial designs. However, outcome selection for such trials can prove challenging. The objectives of this review were to:

(i) Identify and map core outcome sets (COS), across 11 immune-mediated inflammatory diseases (IMIDs) in order to facilitate the selection of relevant outcomes across the conditions for innovative trials of tissue-agnostic drug therapies.
(ii) Compare outcomes or endpoints recommended by the US Food and Drug Administration (FDA) and European Medicines Agency (EMA) to identify and highlight similarities and differences.

**Methods:**

The Core Outcome Measures in Effectiveness Trials (COMET), International Consortium for Health Outcomes Measurement (ICHOM), FDA and EMA databases were searched from inception to 28th December 2019. Two reviewers independently screened titles and abstracts of retrieved entries and conducted the subsequent full text screening. Hand searching of the reference lists and citation searching of the selected publications was conducted. The methodological quality of the included peer-reviewed articles was independently assessed by the reviewers based on the items of the COS–Standards for Development recommendations (COS–STAD) checklist. Core outcomes from the included publications were extracted and mapped across studies and conditions. Regulatory guidance from FDA and EMA, where available for clinical trials for the IMIDs, were obtained from their databases and recommendations on outcomes to measure directly compared.

**Results:**

Forty-four COS publications were included in the final analysis. Outcomes such as disease activity, pain, fatigue, quality of life, physical function, work limitation/productivity, steroid use and biomarkers were recommended across majority of the conditions. There were significant similarities and differences in FDA and EMA recommendations. The only instance where either regulatory body directly referenced a COS was for jSLE—both referenced the Paediatric Rheumatology International Trials Organization (PRINTO) COS.

**Conclusions:**

The findings from this systematic review provide valuable information to inform outcome selection in tissue-agnostic trials for IMIDs. There is a need for increased collaboration between regulators and COS developers and inclusion of regulators as key stakeholders in COS development to enhance the quality of COS.

**Trial registration:**

Not registered.

**Supplementary Information:**

The online version contains supplementary material available at 10.1186/s13063-022-06000-w.

## Introduction

Immune-mediated inflammatory diseases (IMIDs) such as rheumatoid arthritis (RA) and Sjogren’s syndrome (SS) belong to a group of chronic and highly disabling inflammatory conditions [1]. Recent findings that these clinically dissimilar diseases share similar immune dysregulation and molecular drivers of inflammation has sparked an interest in the development of novel therapies that may be used across inflammatory diseases regardless of the specific diagnosis [2, 3]. Selection for such targeted treatment would be based on patients’ response to the novel drugs which would be determined by these molecular drivers [3].

This innovative approach mirrors the new ‘tissue-agnostic’ drug development paradigm in oncology where targeted therapies are developed based on molecular markers rather than organ or tissue type [4, 5]. Tissue-agnostic drug development has already shown considerable promise in oncology with the FDA granting accelerated approvals for drugs such as Keytruda (pembrolizumab) and Vitrakvi (larotrectinib) for the treatment of solid tumours [6]. The EMA recently granted the conditional approval of Vitrakvi [7].

Tissue-agnostic therapies in IMIDs may be evaluated in innovative clinical trials such as biomarker-adaptive and basket trials. Biomarker-adaptive trials incorporate adaptive clinical trial methodology to modify the trials according to the accumulating outcome data [8]. In basket trials, patients are primarily grouped according to molecular drivers rather than their specific diagnosis [9, 10]. The expectation is that group sensitivities to the therapies can be assessed and compared and populations most likely to benefit from treatment identified [9, 11]. The use of basket trials have increased over the past 5 years and is set to increase rapidly over the next few decades as it becomes more widely adopted [12].

To facilitate cross-disease comparisons, it is essential that trial data from the patient groups are comparable. However, at present, a wide variation exists in the outcomes, endpoints and measures selected for use in drug trials. It should be noted that there is a distinction between the terms ‘outcome’ and ‘endpoint’ [13]. According to the NIH Collaboratory ‘….outcome usually refers to the measured variable (e.g. peak volume of oxygen (VO2) or PROMIS Fatigue score), whereas an endpoint refers to the analysed parameter (e.g. change-from-baseline at 6 weeks in mean PROMIS Fatigue score)….’ [13] The variations in outcomes and endpoints measured in trials make it difficult to compare and/or synthesise outcome data within and across IMIDs [14]. As a result, there may be variations in the trial data submitted to support applications for drug approvals and health technology assessment making head-to-head comparisons of drug efficacy and cost-effectiveness analyses challenging.

Core outcome sets (COS), which propose a minimum set of outcomes to measure and report for all trials in specific condition(s), have been developed to assist with the standardisation of outcomes measured in clinical trials [14, 15]. However, there may be variations in the COS proposed for different IMIDs and by different organisations due to differing foci and interests. There is therefore a need to identify appropriate outcomes and endpoints to measure across IMIDs in innovative tissue-agnostic trials.

The Birmingham National Institute for Health Research (NIHR) Biomedical Research Centre for Inflammation was founded to improve healthcare for patients with chronic immune-mediated inflammatory diseases, by developing and accelerating access to new diagnostic tests and new therapies. A programme of observational and experimental clinical trials will be undertaken to achieve this focusing on several IMIDs. The target IMIDs include the following: (i) rheumatoid arthritis (RA); (ii) juvenile idiopathic arthritis (JIA); (iii) ankylosing spondylitis (AS); (iv) psoriatic arthritis (PsA); (v) Sjogren’s syndrome (SS); (vi) Crohn’s disease (CD); (vii) ulcerative colitis (UC); (viii) uveitis (Uv); (ix) systemic lupus erythematosus (SLE) including juvenile SLE (jSLE); (x) autoimmune hepatitis (AIH); and (xi) primary sclerosing cholangitis (PSC). This review therefore focuses on these 11 IMIDs.

The specific objectives were:

(i) Identify and map core outcome sets (COS), across 11 IMIDs in order to facilitate the selection of relevant outcomes across the conditions for innovative trials of tissue-agnostic drug therapies.
(ii) Compare outcomes or endpoints recommended by the US Food and Drug Administration (FDA) and European Medicines Agency (EMA) to identify and highlight similarities and differences.

## Methods

This study was conducted in compliance with the Preferred Reporting Items for Systematic Reviews and Meta-Analysis (PRISMA) guidelines [16] (see PRISMA checklist). Ethical approval was not required for this study as it did not use patient data.

Two reviewers (OLA, LFR) systematically searched from inception to 28th December 2019 four online resources namely the (i) Core Outcome Measures in Effectiveness Trials (COMET), (ii) International Consortium for Health Outcomes Measurement (ICHOM), (iii) European Medicines Agency (EMA) and (iv) US Food and Drug Administration (FDA) databases.

### Search strategy

The search on the COMET database [17] was restricted by selecting 23 relevant disease terms from the ‘disease name’ menu (Additional file 1). The other databases did not have this function therefore the 11 disease terms listed above were entered directly into their search boxes. Guidance documents were obtained by specifically searching the ‘Guidance, Compliance, & Regulatory Information’ section of the FDA [18] and the ‘Scientific Guidelines, the Clinical Efficacy and Safety Guidelines’ section of the EMA website [19].

### Selection of publications

Studies archived on the COMET database were eligible that reported preliminary or definitive COS and outcome measures established through ranked consensus-based methodologies [14]. Purely methodological studies, COS study protocols, reviews of outcomes, outcome measures or symptoms which do not report a consensus-based approach were excluded. Articles reporting COS developed for trials of non-pharmaceutical interventions were also excluded. Published COS from the ICHOM and regulatory guidance provided by the EMA and FDA databases were eligible if focussed on the conditions of interest.

Initial screening of all titles and abstracts was independently conducted by the reviewers (LFR, OLA). The full texts of publications potentially meeting the eligibility criteria were obtained and independently reviewed by the same reviewers. The reasons for exclusion at this stage were documented. At each stage, disagreements regarding eligibility were resolved through discussion and, if necessary, consultation with a third reviewer (MC). Reasons for exclusion were recorded. We conducted a hand search of reference lists and citation search of the included publications.

### Quality assessment

The methodological quality of the included studies peer-reviewed articles was independently assessed by the reviewers based on the items of the COS–Standards for Development recommendations (COS–STAD) checklist [20]. Differences in assessments were discussed and resolved.

### Data extraction strategy

An electronic form was designed, piloted and used for data extraction by the reviewers (LFR, OLA). Data from all the COS publications (peer-reviewed COS articles and the regulatory guidance documents) were extracted verbatim by the two reviewers and cross-checked by a member of the research team (AR) for accuracy. Where available, the reviewers extracted information on:

(i) Recommended core outcomes, endpoints and measures.
(ii) Target patient populations (age, gender, inflammatory condition(s)), study design (e.g. interviews, focus groups, Delphi), contributing stakeholders (e.g. patients, healthcare professionals, carers), geographical location of stakeholders and setting for COS use.
(iii) Methods used to derive, prioritise and/or select the final list of COS, endpoints and/or measures.

### Data synthesis and presentation

The extracted outcomes and endpoints from the COS articles and regulatory guidance documents were grouped by OLA and LFR into sub-domains and domains based on their classification by the source publications. Where there were discrepancies in the domain and sub-domain classifications by different publications, the reviewers discussed and chose the most appropriate for this study. The reviewers inductively grouped domains into broad categories after completing data extraction based on characteristics of the domains.

A matrix was created for each condition displaying the core outcomes, endpoints and any recommended outcome measures extracted from each publication. Publications were arranged according to the COS’s target study design (e.g. longitudinal, clinical trial). These matrices were combined to form a single matrix showing all core outcomes, endpoints and measures recommended across all inflammatory conditions.

For the second study objective, the extracted outcomes, endpoints and measures recommended by the FDA and EMA in scientific guidance documents were separately compared to highlight similarities and differences. The findings were presented in a table. The matrix and the final tables were cross-checked by AR for accuracy.

## Results

### Characteristics of included publications

**Fig. 1:**
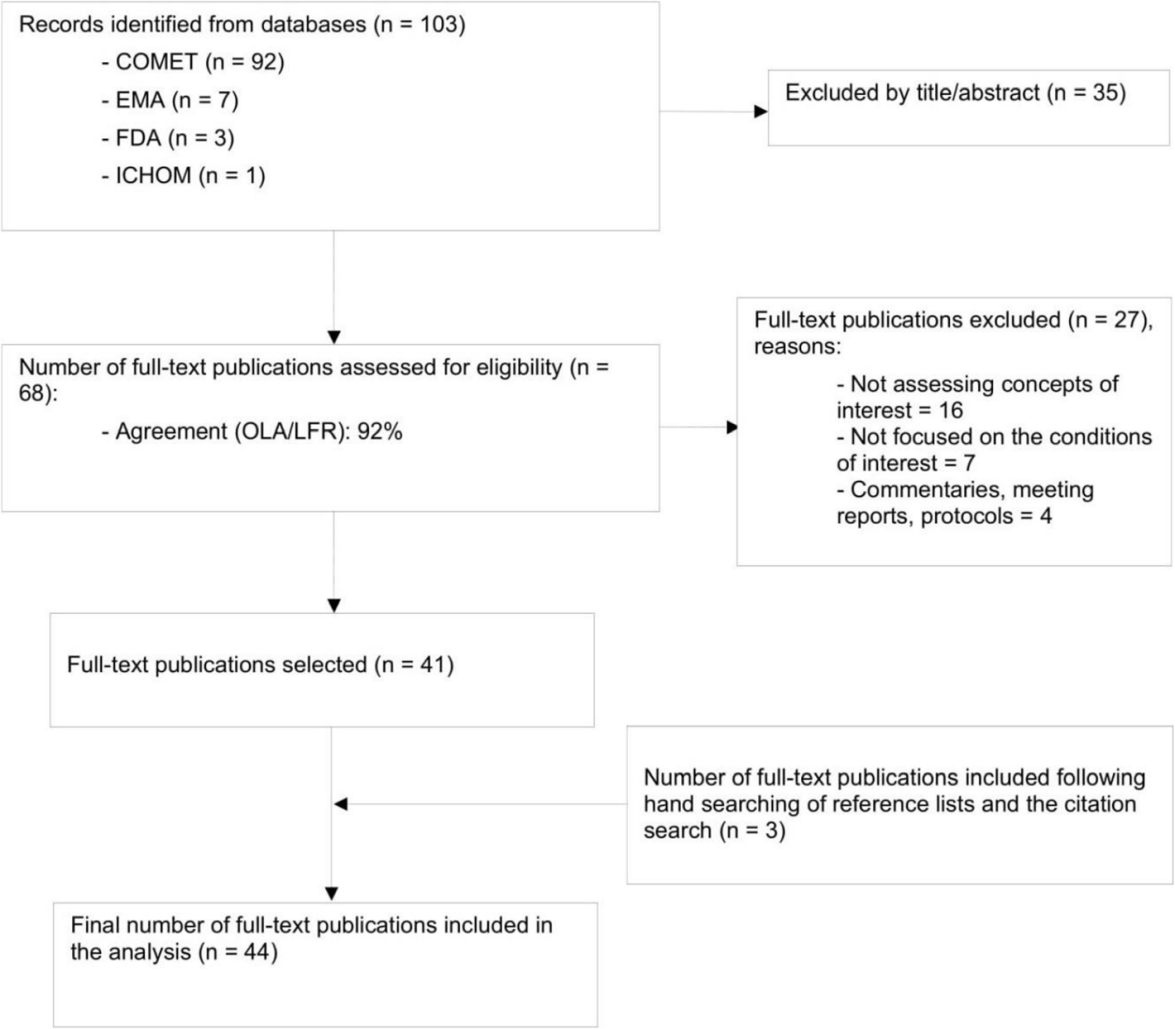
PRISMA flow diagram

The selection process is depicted in a PRISMA flow diagram (see Fig. 1). Table 1 summarises the 44 included publications (peer-reviewed COS articles and regulatory guidance documents) and provides details on the characteristics of the included publications [21–64]. See Supplementary Table 2 for further details.

**Table 1:** Characteristics of included publications

Characteristics	Inflammatory conditions									
	ALL	RA*	JIA	AS	PsA	SS	CD**	UC	Uv	SLE*** (jSLE)
Total number included	44	10	2	4	8	2	7	2	1	6 (2)
Type of publication:										
Collaborative reports	34	8	1	3	7	2	6	0	1	4 (2)
Regulatory guidance	10	2^X^	1^e^	1^e^	1^e^	0	1^e^	2^X^	0	2^X^ (0)
Patient population:										
Adults	37^ap^	10^ap^	–	4	8	2	5^ap^	2^ap^	0	6^ap^
Paediatric specific	7	0	2	0	0	0	2	0	1	2
Purpose:										
Trials and LOS	43	10	2	3	8	2	7	2	1	6 (2)
Routine practice	8	2	0	2	2	0	1	0	0	0 (0)
Registries	4	0	0	3	1	0	0	0	0	0 (0)
Methods:										
Initial literature or systematic review/database search	22	6	0	3	3	1	5	0	1	3 (0)
Surveys	9	2	1	1	2	1	0	0	1	0 (1)
Interviews/focus groups	4	0	0	1	2	0	1	0	0	0 (0)
Delphi/ NGT/other consensus group meeting	32	8	1	2	6	2	6	0	1	4 (2)
Patient involvement	14	5	0	2	4	0	3	0	0	0 (0)
HCP involvement	34	8	1	3	7	2	6	0	1	4 (2)

*AS* ankylosing spondylitis, *CD* Crohn’s disease, *EMA* European Medicines Agency, *FDA* Food and Drug Administration, *HCP* health care professionals, *ICHOM* International Consortium for Health Outcomes Measurement, *JIA* juvenile idiopathic arthritis, *LOS* longitudinal observation studies, *NGT* nominal group technique, *PsA* psoriatic arthritis, *RA* rheumatoid arthritis, *SLE* systemic lupus erythematosus, *jSLE* juvenile SLE, *SS* Sjogren’s syndrome, *UC* ulcerative colitis, *uV* uveitis

*ICHOM 2018 document for inflammatory arthritis covers RA, PsA, AS and JIA

**Kim 2018 and Ruemmele 2014 referred to both CD and UC (inflammatory bowel disease). To avoid confusion, these have been recorded under CD

***Values in brackets represent the subsets relating to jSLE (under SLE)

^e^Denotes an EMA publication whilst ^f^ represents an FDA document

^x^Signifies that both EMA and FDA produced documents for the condition

^ap^Signifies the inclusion of publications with mixed patient populations

#### Number of publications included

COS were found for all conditions except AIH and PSC. A total of 92 publications from COMET were screened and of these 30 were included [22–25, 27, 28, 30, 31, 33, 35, 37–42, 45, 46, 48–51, 54–56, 58–61, 64]. EMA guidance for AS, CD, JIA, PsA, RA, SLE and UC [21, 26, 32, 34, 43, 52, 62], FDA guidance for RA, SLE and UC [44, 53, 63] and one document from the ICHOM database [47] were included. No regulatory guidance was found for Uv or SS. Following reference list and citation searching, three articles [29, 36, 57] were retrieved bringing the total number of publications included in the final analysis to 44 [21–64]. RA had the highest number of relevant publications (10 in all) whilst the only publication included for uveitis actually relates to JIA-related uveitis [64].

#### Study populations and settings

In terms of study populations, thirty-seven publications were associated with COS for adults or mixed populations [21–26, 28–30, 34–57, 60–63] whilst seven related to COS specifically for paediatric patients [27, 31–33, 58, 59, 64].

All the COS were designed/recommended for use in clinical trials and longitudinal observational studies (with the exception of the COS specifically developed for AS registries by Zochling et al. [24]). Five of these COS were also recommended for use in routine clinical practice for AS [23], perianal CD [28], inflammatory bowel disease (IBD) [30], PsA [35] and RA [50]. The ICHOM COS was developed for use in clinical trials and routine practice for all ‘inflammatory arthritis’ [47].

### Quality assessment

#### Consensus methods used

All the COS articles reported employing consensus methods—nine studies (28%) used Delphi/modified Delphi methods, 11 (34%) used nominal group technique and 12 (38%) employed unspecified consensus methods. The included articles generally provided adequate information pertaining to scope (COS-STAD items 1–4).

#### Stakeholder involvement

Information relating to stakeholder involvement (COS-STAD items 5–7) and consensus process (COS-STAD 8–11) were sometimes less detailed making it difficult to assess the degree of stakeholder involvement and the robustness of the consensus process. Whilst clinical experts were involved in the COS development process for 34 studies, only 14 publications explicitly reported patient involvement, with one reporting that patients were not involved [64]. Details about the characteristics of panels or working groups were often limited making it difficult to ascertain the inclusion of patients and their specific involvement. The period COS were developed seemed to influence the reporting of patient involvement. COS published within the last decade (such as Nikiphorou et al. [49] and Radner et al. [50] for RA; Orbai et al. [39] and Tillett et al. [41] for PsA) were more likely to report patient involvement explicitly than older ones (such as Felson et al. for RA [45] and Gladman et al. [37] for PsA). However, we were unable to rule out the possibility that the developers of some of the older COS might have involved patients to some degree but the authors have not reported this in their publication. See Additional file 2 for further details. It should be noted that the regulatory guidance documents did not report the use of any consensus process or stakeholder involvement to inform the recommendations provided.

### Core outcomes proposed across the inflammatory conditions

#### Core outcomes proposed

Table 2 summarises the core outcomes extracted from the COS articles and the regulatory guidance documents across the nine included inflammatory conditions. Outcomes such as disease activity, joint/structural damage, pain, fatigue, quality of life, physical function, work limitation/productivity, steroid use and biomarkers (acute phase reactants) were recommended across majority of the conditions. Psychosocial function, psychological and emotional wellbeing were the least frequently recommended ‘generic’ outcomes across the conditions. Expectedly, outcomes such as rectal bleeding which is specific to UC and sicca symptoms which relate to SS had very low frequencies.

**Table 2:** Core outcomes proposed across inflammatory conditions

Category	Domain	Sub-domain/group	RA [50]	AS [22]	PsA [39]	SS [60]	CD [30]	UC [30]	SLE [56]	jSLE	JIA	Uv [64]	Sum
Disease activity													
	Patient global assessment of wellbeing		✓^†^	✓*^†^	✓*^†^					✓*^†^	✓*		5
	Clinician global assessment of disease activity		✓^†^		✓*			✓*	✓	✓*^†^	✓*		6
	Disease activity		✓*	✓		✓^†^	✓*^†^	✓*^†^	✓*^†^~	✓*^†^	✓*		8
		Low disease activity	✓*										1
	Musculoskeletal (MSK) disease activity	Peripheral joints		✓*^†^	✓*^†^								2
		Enthesitis		✓*^†^	✓*^†^						✓		3
		Dactylitis			✓*^†^						✓		2
		Spine symptoms			✓*^†^								1
	Tender joints		✓*^†^	✓									2
	Swollen joints		✓*^†^	✓*									2
	Joint or structural damage		✓*^†^	✓*^†^	✓*						✓	✓^†^	5
	Organ damage					✓^†^			✓*^†^~	✓*			3
	Systemic inflammation				✓*^†^								1
	Ocular surface damage					✓							1
	Visual acuity											✓^†^	1
	Grade of cells in anterior chamber											✓^†^	1
	Grade of flare in anterior chamber											✓^†^	1
	Flares								✓*				1
	Comorbidities		✓^†^										1
	Biomarkers	Auto-antibody status	✓^†^			✓				✓^†^			3
		Anti-drug antibody						✓*					1
		Acute phase reactants	✓*^†^	✓*^†^	✓*		✓*	✓*			✓*		6
		Unspecified							✓			✓^†^	2
	Laboratory indices									✓*^†^			1
	MAS										✓		1
	Skin disease activity	Skin			✓*^†^								1
		Nails			✓*^†^								1
		Itching			✓^†^								1
	Perianal disease activity												
		Dev of perianal abscess					✓						1
		Dev of a new/recurrent fistula					✓						1
		Unplanned surgical intervention					✓						1
		Faecal diversion/ proctectomy					✓						1
Clinical endpoints	Mucosal healing (endoscopic)						✓*~	✓*					2
	Histological evaluation of mucosal inflammation							✓*					1
	Symptomatic remission						✓*	✓*					2
	Clinical remission		✓*^†^				✓*^†^~	✓^†^*					3
	Remission without steroid						✓*~	✓*					2
	Complete clinical response						✓~						1
	Sustained clinical benefit						✓~						1
	Radiological remission						✓~						1
	Radiological response						✓~						1
	Deep remission						✓~						1
	Occlusive symptoms (absence)						✓~						1
	Endoscopic remission						✓~						
	Bowel damage progression						✓~						1
	Treatment and therapeutic failure						✓~						1
	Long term efficacy						✓~						1
	Steroid free						✓~						1
	Clinical success/benefit						✓~						1
	Fistula healing						✓*						1
	Time to remission						✓*	✓*					2
	Time to response						✓*	✓*					2
	Clinical assessment of drainage						✓*						1
	Salivary flow					✓							1
	Ophthalmic outcome					✓							1
	Symptom free survival						✓~						1
	Overall survival						✓^†^	✓^†^					2
	Colorectal cancer						✓^†^	✓^†^					2
Symptoms, QOL, function													
	Pain		✓*^†^	✓*^†^	✓*^†^		✓^†^	✓^†^			✓*		6
	Anaemia						✓^†^	✓^†^					2
	Morning stiffness										✓*		1
	Spinal stiffness and mobility			✓*^†^									1
	Fatigue		✓*	✓*^†^	✓^†^	✓	✓^†^	✓^†^	✓*		✓		8
	Fever										✓		1
	HRQOL (Generic)		✓^†^	✓*	✓*^†^	✓^†^	✓		✓*^†^~	✓*	✓	✓^†^	9
	HRQOL (Specific)			✓*	✓*^†^	✓	✓*~	✓*	✓*~				6
	Physical function/Disability		✓*^†^	✓*^†^	✓*^†^		✓^†^~	✓^†^~	✓*			✓^†^	7
	Function (General)		✓*			✓^†^					✓*		3
	Psychosocial function		✓				✓					✓	3
	Psychological health/emotional well being		✓		✓		✓^#^	✓					4
	Sexual activity						✓^#^						1
	Overall control						✓	✓					2
	Work limitation/productivity		✓	✓	✓		✓^#^		✓*				5
	Sicca symptoms	Dry eyes				✓^†^							1
		Dry mouth				✓^†^							1
	Change in bowel symptoms						✓^†^	✓^†^					2
	Rectal bleeding							✓*					1
	Stool frequency							✓*					1
	Stool consistency							✓					1
	Impact of fistula						✓	✓					2
	Global assessment of incontinence						✓						1
Healthcare utilisation													
	Surgery		✓				✓~	✓				✓	4
	Reduction in surgical procedures						✓*						1
	Time spent/number of hospital visits						✓^†^	✓^†^				✓^†^	3
	DMARD use		✓^†^								✓		2
	Steroid use		✓^†^				✓^†^	✓*^†^	✓*~			✓	5
	Non-drug treatments		✓										1
Others													
	SAE/safety outcomes					✓		✓*^†^	✓^†^~		✓		4
	Toxicity					✓			✓^†^				2
	Death/cause of death		✓					✓	✓~				3
	Cost/Cost-effectiveness					✓							1
	Weight		✓^†^				✓^†^	✓^†^					3
	Nutritional status						✓	✓					2
	Disease duration		✓^†^										1
	Smoking		✓^†^										1
Paediatric-specific													
	Parent global assessment of disease activity							✓		✓^†^			2
	Growth						✓*	✓		✓^†^	✓		4
	Improved growth pattern						✓*						1
	Normalised growth						✓*						1
	School absence											✓^†^	1
	Extra-intestinal manifestations						✓*						1

*AS* ankylosing spondylitis, *CD* Crohn’s disease, *DMARDs* disease-modifying anti-rheumatic drugs, *HRQOL* health-related quality of life, *JIA* juvenile idiopathic arthritis, *MAS* macrophage activation syndrome, *PsA* psoriatic arthritis, *RA* rheumatoid arthritis, *SAE* serious adverse event, *SLE* systemic lupus erythematosus, *jSLE* juvenile SLE, *SS* Sjogren’s syndrome, *UC* ulcerative colitis, *uV* uveitis

*Suggested by EMA and/or FDA

†Proposed as part of core outcome set by non-regulatory research groups

# It was suggested that these 3 domains along with ‘Lifestyle restriction based on toileting needs’ be combined to give a ‘patient priorities’ score (Sahnan 2018)

~ Proposed as critical or important endpoints for CD by Danese et al. [25] or suggested as endpoints for SLE by Gordon [54]

Definitions

Symptomatic remission—complete absence of occlusive symptoms—abdominal pain and/or nausea and/or vomiting and/or bloating and/or diet restriction after meals (Danese 2018)

Sustained clinical benefit—no additional treatment and daily life nearly symptom-free, or additional treatment (except surgery) with good function in society

Radiological remission—bowel wall thickness (< 3 mm), bowel dilation (diameter < 25 mm). Bowel stricture (diameter > 10 mm)

Deep remission—complete mucosal healing and clinical/biochemical remission (defined as HBI score < 5 ± CRP < 5 mg/L or calprotectin < 50 mg/g)

Treatment failure - Any CD-related surgery, or hospitalisation, or penetrating complication, or need for corticosteroids or biological drug

Therapeutic failure - CD-related surgery, or drug discontinuation because of lack of efficacy, or loss of response, or failure to respond to dose escalation or intolerance, or drug switched to another drug because of inadequate response/loss of response

Fistula healing - Closure and maintenance of closed fistula without development of new fistulas or abscesses

#### Approach to outcome recommendations

One of the issues identified by this review was the difference in approach by the various COS developers and regulatory bodies. Regulatory bodies often suggested a list of ‘primary’ and ‘secondary’ endpoints from which trialists may make selections. On the other hand, COS developers propose a minimum set of outcomes (core items) to be measured in trials, sometimes complemented by optional or ‘outer core’ items [39].

#### Terminological inconsistencies

Another observation was the inconsistency in terminologies used by both regulatory bodies and COS developers. For example, the study by Heijde et al. [22] used the terms ‘measures’, ‘endpoints’ and ‘domains’ interchangeably to refer to outcomes such as pain and physical function [22]. Similarly, the 2015 EMA guidance for SLE [52] used the terms ‘outcomes’ and ‘endpoints’ interchangeably. The report stated ‘primary outcomes’ before going on to discuss ‘secondary endpoints’ [52].

#### Differences in recommendations

There were sometimes differences in the recommendations by regulatory bodies and COS developers. For example, the 2018 EMA guidance for Crohn’s disease [26] recommended fistula healing (demonstrated by MRI) as the primary endpoint for fistulising perianal Crohn’s disease whilst the COS developed by Shahan et al. [28], for the same population, included fistula response on MRI as optional [28].

There were disparities in recommendations for the use of biomarkers as outcomes or measures, with some studies cautioning against their use in specific patient subpopulations or disease stages. For instance, Ruemmele et al. [31] noted that C-reactive protein (CRP) is not elevated in all patients with active Crohn’s disease, limiting its usefulness, and although superior to CRP, faecal calprotectin has large variability in results and low responsiveness [31].

### Outcome measures proposed across the target IMIDs

Outcome measures proposed across the target IMIDs can be found in Additional file 3.

#### Availability of outcome measures

It was observed that COS research groups tended to focus initially on achieving consensus and publishing their COS before commencing work on outcome measures to recommend in subsequent publications. For instance, Heijde et al. only reported the COS for AS in their initial article in 1997 [22]. However, 2 years later they published their work on outcome measures [23]. A similar scenario was observed with PsA where an earlier paper authored by Gladman et al. for the OMERACT PsA Working Group only reported COS [35] whilst a subsequent article presented outcome measures [36]. The latest publication from the group reported an update of the PsA COS and intimated that a thorough investigation of available measures would be commenced [38].

#### Information about validity of outcome measures

Although the COS studies suggested outcome measures to measure majority of the proposed COS, there was patchy information about the validity of these measures. Whilst the regulatory guidance and a few studies such as Gladman [36] explicitly discussed the available evidence of the validity of the measures proposed, the majority of the studies did not. Therefore, the basis of their recommendations was unclear, and this might explain the heterogeneity that was found in the recommendations.

### Comparison of FDA and EMA recommendations

We were only able to compare FDA and EMA recommendations extracted for RA, SLE, jSLE and UC as these were the only conditions that had published FDA guidance documents. The findings are presented in Table 3.

**Table 3:** Comparison of FDA and EMA guidance

	FDA	EMA
RA	FDA 2013 guidance [65] Key RA domains: (i) Clinical response: ACR20 to demonstrate reduction in disease activity. Supportive evidence of efficacy: (a) higher levels of response, measured by ACR50, ACR70 (b) measures of low disease activity (LDA): DAS28 (ii) Improvement in physical function: HAQ-DI Other domains: (i) Prevention of structural damage progression: Radiographic data using validated scoring methods. (ii) (ii) Clinical remission: ACR/EULAR Provisional Definition of Remission criteria may be acceptable.	EMA 2017 guidance [66] Primary endpoint(s): (i) Remission (3–6 months): by a combined measure (studies on the treatment of naïve patient) (ii) LDA (3–6 months): In patients with inadequate response to synthetic or biologic DMARD treatment: DAS28-CRP, DAS28-ESR, SDAI or CDAI Secondary endpoints: (i) ACR20, ACR50, ACR70 responder rates (ii) Structural joint damage by X-rays (e.g. Sharp-van der Heijde scores) (iii) Physical function (e.g. HAQ-DI) (iv) Remission/LDA rates defined by SDAI, CDAI, DAS28-ESR or-CRP (if not already not chosen as primary endpoint) Others: (i) CRP (ii) Pain: VAS or Numeric Rating Scale (iii) Quality of Life: SF-36, AIMS (iv) Fatigue: FACIT-F (v) Ultrasonography of the joints (vi) MRI of the joints (RAMRIS scale)
SLE	FDA 2010 guidance [68] Primary Efficacy Endpoints: (i) Reduction in disease activity: BILAG is the preferred index. SLEDAI, SELENA-SLEDAI, SLAM, ECLAM. The primary efficacy analysis can be based on the outcome of major clinical response (MCR) or partial clinical response (PCR) (ii) Complete clinical response or remission (iii) Reduction in flare/increase in time to flare (iv) Reduction in concomitant steroids (v) Treatment of serious acute manifestations Secondary endpoints: (i) PRO instruments: No existing PRO instrument was considered optimal for measurement of fatigue symptom complex. Others: (i) An assessment of damage caused by manifestations of SLE least 1-year duration (SLICC/ACR Damage Index measures) (ii) Biomarkers	EMA 2015 guidance [67] Primary outcomes: (i) Control of the disease activity (SLEDAI and BILAG, SLE Responder Index [SRI] or BICLA (ii) Prevention of flares (Criteria for flares should be predetermined in the protocol: using SLEDAI-2 K, SELENA-SLEDAI, BILAG score; time to a new flare or the frequency/annual rate of flares) (iii) Prevention of long-term damage (the SLICC/ACR damage index, clinical trial should be at least 12 months) Secondary endpoints: (i) When a composite endpoint is used as a primary outcome measure components of this composite endpoint should be analysed separately as secondary outcomes (ii) Decrease in steroid dose (iii) Patients and investigators reported outcomes: (a) HRQOL – SF-36 and any of: Lupus QoL, SLE symptom checklist, SLE QOL; WPAI Lupus score; FSS; FACIT-F or BFI; ADL for change in physical function (iv) Biomarkers
jSLE	FDA SLE 2010 [68] referencing PRINTO core set of domains: (i) A DAI: e.g. ECLAM, SLEDAI, SLAM, BILAG (ii) Renal function: 24-h proteinuria (iii) Parent’s global (iv) Physician’s global (v) Health status: CHQ physical summary score	EMA 2015 [67] referenced the PRINTO domains: (i) Physician’s global assessment of disease activity (ii) A global DAI (e.g. ECLAM, SLEDAI, SLAM, BILAG (iii) 24-h proteinuria. Alternatively, the spot urine protein: creatinine ratio on first morning void urine sample (iv) Patient’s/Parent’s global assessment of the overall patient’s wellbeing (v) HRQOL: CHQ physical summary score
UC	FDA 2016 [70] Primary endpoints: (i) Clinical remission (responder definition based on stool frequency, rectal bleeding and endoscopy scores). This is the recommended one. Until a valid PROM for UC signs and symptoms and a valid clinician rating scale for mucosal inflammation in UC become available, a modified Mayo or modified UCDAI score (omitting the physician’s global or disease activity ratings) can be used as an endpoint measure. Secondary endpoints: (i) Changes between the treatment arms of each of the subscores (Stool Frequency, Rectal Bleeding and Endoscopy) (ii) And/or the total score (i.e. sum of the Stool Frequency, Rectal Bleeding and Endoscopy subscores). (iii) Corticosteroid-free remission (based on a justified minimum duration of time over which a patient is considered to be both corticosteroid-free and in clinical remission) (iv) Endoscopic Appearance of the Mucosa - There are currently limitations of histologic scoring systems and of community standards for definitions of histologic improvement; thus, there are currently no criteria for histological assessment of mucosal healing.	EMA 2018 [69] Stressed that the total Mayo score including physician’s global assessment is not of primary interest. Primary endpoint: (i) Proportion of patients with symptomatic remission (ii) Proportion of patients with endoscopic remission Secondary endpoints: (i) Patients achieving both MH and symptomatic remission (ii) Patients achieving response: Response should be defined according to the instruments used for evaluating symptoms and endoscopic appearance. (iii) Patients achieving remission defined more stringently than for the primary endpoint or vice versa (iv) In studies where steroids are not tapered at time of evaluation of the primary endpoint, (a) proportions of patients in whom either or both symptomatic and endoscopic remission are achieved without concomitant steroid treatment (b) proportions of patients in whom either or both symptomatic and endoscopic remission are achieved at particular doses of concomitant steroid treatment (v) Numerical, separate evaluations of the individual components of the symptom score and of MH score (vi) Histological evaluation of mucosal inflammation, including number of patients achieving histological normalisation (vii) Individual patients achieving MH, judged endoscopically, as well as combined symptomatic, biomarker and histological normalisation (viii) Changes in stool frequency (ix) Laboratory measures of inflammation (e.g. faecal calprotectin) (x) Time to remission (symptom scores and biomarkers only) (xi) Time to response (symptom scores and biomarkers only) Other secondary endpoints: (xii) Validated QoL measurement, e.g. inflammatory bowel disease questionnaire (IBDQ) (xiii) Reduction in number of colectomies (primarily relevant in studies of acute severe ulcerative colitis).

*Lupus QoL* Lupus Quality of Life, *SLE QoL* SLE symptom checklist and SLE Quality of Life, *WPAI* Work Productivity and Activity Impairment Lupus score, *FSS* fatigue severity scale, *BFI* FACIT fatigue or the Brief Fatigue Inventory, *ECLAM* ADL for change in physical function. European Consensus Lupus Activity Measure, *SLEDAI* Systemic Lupus Erythematosus Disease Activity Index, *SLAM* Systemic Lupus Erythematosus Activity Measure, *BILAG* British Isles Lupus Assessment Group, *DAI* disease activity index, *CHQ* Child Health Questionnaire, *IBDQ* inflammatory bowel disease questionnaire, *UCDAI* Ulcerative Colitis Disease Activity Index, *HAQ-DI* Health Assessment Questionnaire Disability Index, *ACR* American College of Rheumatology

#### Comparison of guidance for RA

Comparing the FDA 2013 guidance for RA [65] with the corresponding EMA 2017 document [66], there were three key differences. Whilst the FDA regards clinical response measured by the ACR20 as a key domain for RA, and clinical remission as a secondary domain, the EMA considers clinical remission as a primary endpoint and does not recommend improvement in measures such as ACR20 as primary endpoints as their ‘clinical relevance may not be immediately clear’. [65, 66] In addition, the FDA guidance considered improvement in physical function as a key domain to assess whilst the EMA considered it as a secondary endpoint [65, 66]. However, both recommended the HAQ-DI for the assessment of physical function [65, 66].

#### Comparison of guidance for SLE

The FDA 2010 guidance for SLE and the corresponding EMA 2015 guidance considered the assessment of disease activity index (DAI), and reduction in flares as primary endpoints [67, 68]. Similar measures including the BILAG, SLEDAI, SLAM and ECLAM were recommended by both regulatory bodies to assess these two endpoints [67, 68]. However, whilst the FDA guidance regarded a reduction in concomitant steroids as a primary endpoint and the assessment of damage as a secondary endpoint, the order was reversed in the EMA guidance. Both documents recommended that the SLICC/ACR Damage Index is used to assess damage over a minimum period of 12 months [67, 68]. The FDA opinion was that there were no optimal measures for fatigue and so did not recommend any PRO measures [68]. On the other hand, the EMA recommended combining the SF-36 with any of the SLE-specific measures and also the FACIT-F or the BFI for the assessment of fatigue [67].

Interestingly, the FDA and EMA recommendations for jSLE matched well as both referenced the Paediatric Rheumatology International Trials Organization (PRINTO) COS [59, 67, 68]. This was the only instance where either regulatory body directly referenced a COS.

#### Comparison of guidance for UC

The key difference between the FDA and EMA guidance for UC was their position on the use of endoscopic remission as a primary endpoint/outcome [69, 70]. The FDA stated that ‘there are currently limitations of histological scoring systems and of community standards for definitions of histological improvement; thus, there are currently no criteria for histological assessment of mucosal healing’ and recommends endoscopic remission as a secondary endpoint [70]. On the other hand, the EMA considered the proportion of patients with endoscopic remission as a primary endpoint [69]. Both regulatory bodies felt there were issues with using the total Mayo score due to the inclusion of physician’s global assessment [69, 70]. The FDA suggested using a modified Mayo or modified UCDAI score (omitting the physician’s global) whilst the EMA stated that the total Mayo score ‘is not of primary interest’. [69, 70] Again the FDA did not consider any PROM as suitable for evaluating the signs and symptoms of UC whilst the EMA recommended validated PROMs such as the IBDQ as a secondary endpoint [69, 70].

## Discussion

This systematic review has identified and mapped, for the first time, existing COS currently recommended for efficacy trials across multiple immune-mediated inflammatory diseases and compared outcomes and/or endpoints recommended by FDA and EMA for similarities and differences.

COS were found for all the conditions except AIH and PSC. The COS found for uveitis was specifically for JIA-related uveitis [64]. Outcomes such as disease activity, joint/structural damage, pain, fatigue, quality of life, physical function, work limitation/productivity, steroid use and biomarkers (acute phase reactants) were recommended across majority of the conditions and should be considered when designing basket trials for tissue-agnostic drug development involving patients with inflammatory diseases. For basket trials, trialists should consider using these common outcomes identified across the conditions in this review as a minimum set and supplement with other outcomes as required for each condition. This will therefore facilitate the comparison of outcomes across IMIDs in basket trials. The review also provides a useful repository of COS for inflammatory diseases and regulatory guidance.

There were significant similarities and differences in FDA and EMA recommendations. The only instance where either regulatory body directly referenced a COS was for jSLE—both referenced the PRINTO COS.

The relatively voluminous literature for some of the conditions, notably for RA and PsA, attests to considerable progress in the recommendation of outcome measures for these conditions. On the other hand, our review highlights the research effort required to produce COS for other conditions, particularly uveitis and Sjogren’s syndrome, for which we found very limited published information.

The differences in approach and inconsistent terminologies used by the regulators and COS developers might explain the disparities we sometimes found in some of the recommendations. Efforts should be made to harmonise the terminologies used by all the organisations. The fact that there was only on instance of the FDA and the EMA directly referencing a COS also indicates the need for increased collaboration across regulators and COS developers and inclusion of regulators in COS development.

Less than half of the COS publications explicitly reported patient involvement and when presented details of this involvement were often vague, with the exception of Tillett et al. [41] The implication of this is that some outcomes included in the COS might not be outcomes meaningful or highly prioritised by patients. The selection of stakeholder relevant outcomes and the need for patient involvement in regulatory decision-making is increasingly recognised as important [71–73].

The main limitation of this study is its reliance on the information explicitly provided in the included publications. For instance, although we noticed a tendency for more recent publications to detail patient involvement in the development of COS, we were unable to rule out the possibility that the developers of some of the older COS might have involved patients to some degree but the authors have not reported this in their publication.

Another limitation of the study is the lack of publications for some conditions such as PSC and the overrepresentation by RA. However, we have ensured that our tables present the results in a manner that clearly reflects this issue. As FDA guidance documents were not available for all the conditions, we were unable to directly compare the recommendations provide by the FDA and EMA for a number of the conditions.

The scope of this review was determined by our programme-specific requirements. Therefore, our findings and conclusions may not be applicable to research that involves a different selection of IMIDs. As the purpose of the review is to facilitate the selection of outcomes across several IMIDs for basket trials, there may be differences between the outcomes recommended in this review and previously published disease-specific COS.

Despite these limitations, by allowing comparison of COS across conditions, this review could facilitate the selection of commonly relevant outcomes that may be measured in tissue-agnostic trials. Measuring the same outcomes across the conditions would demonstrate more accurately the similarities or variations in the response to drug interventions between patient groups. This information could also guide the subsequent recommendations for drug approval. However, further work needs to be done to address the gaps identified especially relating to outcome measures to use in trials. The review highlights the need for greater collaborations between regulatory bodies and COS developers so that stronger and more uniform recommendations can be made which may facilitate the adoption COS. There is also a need for collaboration on the development of COS for routine care which is particularly important for real-world evidence (RWE) generation [74].

## Conclusions

Tissue-agnostic drug development which utilise current advances in precision medicine such as basket trials, have the potential to usher in a new era of drug development in IMIDs. The measurement of a core set of outcomes across the conditions in such trials could facilitate the collection of more robust efficacy data by facilitating direct comparisons between patient groups. This information could potentially improve and strengthen subsequent drug approvals, recommendations and labelling claims. Outcomes such as disease activity, joint/structural damage, pain, fatigue, quality of life, physical function, work limitation/productivity, steroid use and biomarkers (acute phase reactants) should be considered when designing basket trials for tissue-agnostic drug development involving patients with inflammatory diseases. There is a need for increased collaboration between regulators and COS developers and inclusion of regulators as key stakeholders in COS development to enhance the quality of COS.

## Supplementary Information

**Additional file 1:** COMET database search

**Additional file 2:** Characteristics of included publications

**Additional file 3:** Outcome measures proposed across inflammatory conditions

## Data Availability

Relevant data has been provided in tables and additional files.

## Abbreviations

ACR: American College of Rheumatology
AIH: Autoimmune hepatitis
AS: Ankylosing spondylitis
BFI: Brief Fatigue Inventory
BILAG: British Isles Lupus Assessment Group
CD: Crohn’s disease
CHQ: Child Health Questionnaire
COMET: Core Outcome Measures in Effectiveness Trials
COS: Core outcome set(s)
COS-STAD: COS–Standards for Development recommendations
CRP: C-reactive protein
DAI: Disease Activity Index
ECLAM: European Consensus Lupus Activity Measure
EMA: European Medicines Agency
FDA: Food and Drug Administration
FSS: Fatigue severity scale
HAQ-DI: Health Assessment Questionnaire Disability Index
IBD: Inflammatory bowel disease
IBDQ: Inflammatory bowel disease questionnaire
ICHOM: International Consortium for Health Outcomes Measurement
IMIDs: Immune-mediated inflammatory diseases
JIA: Juvenile idiopathic arthritis
jSLE: Juvenile systemic lupus erythematosus
NIHR: National Institute for Health Research
PRINTO: Paediatric Rheumatology International Trials Organization
PRISMA: Preferred Reporting Items for Systematic Reviews and Meta-Analysis
PsA: Psoriatic arthritis
PSC: Primary sclerosing cholangitis
RA: Rheumatoid arthritis
RWE: Real-world evidence
SLAM: Systemic Lupus Erythematosus Activity Measure
SLE: Systemic lupus erythematosus
SLEDAI: Systemic Lupus Erythematosus Disease Activity Index
SLE QOL: SLE symptom checklist and SLE Quality of Life
SS: Sjogren’s syndrome
UC: Ulcerative colitis
UCDAI: Ulcerative Colitis Disease Activity Index
Uv: Uveitis
WPAI: Work Productivity and Activity Impairment
