# Supplementary material for: Outcome selection for tissue-agnostic drug trials for immune-mediated inflammatory diseases: a systematic review of core outcome sets and regulatory guidance
Source: Trials. 2022 Jan 15;23:42. doi: 10.1186/s13063-022-06000-w (PMC8761289; doi:10.1186/s13063-022-06000-w)
Supplement: Supplementary file 1 — Additional file 1: COMET database search [file 13063_2022_6000_MOESM1_ESM.docx]

### **Additional file 1**

### COMET database search

- Health Area - **23** Disease terms (92 matches)
  - Ankylosing spondylitis (AS) (3 matches)
  - Arthritis (4 matches)
  - Axial spondyloarthritis (1 match)
  - Bowel obstruction (1 match)
  - Chronic disease (1 match)
  - Crohn's disease (CD) (6 matches)
  - Inflammatory arthritis (2 matches)
  - Inflammatory bowel disease (IBD) (5 matches)
  - Juvenile arthritis (1 match)
  - Juvenile idiopathic arthritis (JIA) (2 matches)
  - Juvenile systemic lupus erythematosus (JSLE) (2 matches)
  - Lumbar spinal stenosis (1 match)
  - Musculoskeletal conditions (3 match)
  - Nail psoriasis (2 matches)
  - Perianal Crohn's disease (pCD) (1 match)
  - Psoriatic arthritis (PsA) (7 matches)
  - Rheumatic diseases (3 matches)
  - Rheumatoid arthritis (RA) (29 matches)
  - Sjogren's syndrome (SS) (3 matches)
  - Spinal disorders (5 matches)
  - Systemic lupus erythematosus (SLE) (4 matches)
  - Ulcerative colitis (UC) (3 matches)
  - Uveitis (uV) (3 matches)
