# Supplementary material for: Outcome selection for tissue-agnostic drug trials for immune-mediated inflammatory diseases: a systematic review of core outcome sets and regulatory guidance
Source: Trials. 2022 Jan 15;23:42. doi: 10.1186/s13063-022-06000-w (PMC8761289; doi:10.1186/s13063-022-06000-w)
Supplement: Supplementary file 2 — Additional file 2: Characteristics of included publications [file 13063_2022_6000_MOESM2_ESM.docx]

**Additional file 2**

| Characteristics of included publications | | | | | | | | | | | | | |
| --- | --- | --- | --- | --- | --- | --- | --- | --- | --- | --- | --- | --- | --- |
| Study | Affiliation(s) | Condition | Patient group | | Purpose | | | Methods | | | | | |
|  |  |  | Adult | Paediatric | Trials & LOS | Routine practice | Registry/  record keeping | Lit. Review/  Database  search | Surveys | Qualitative work (interviews, focus group) | Delphi/ NGT/Con-sensus  group meeting | Clinician  Involvement | Patient  Involvement |
| EMA 2017^24^ | EMA | AS | ✓ |  | ✓ |  |  |  |  |  |  |  |  |
| Van d. Heijde 1997^25^ | ASAS | AS | ✓ |  | ✓ | ✓ | ✓ | ✓ |  | ✓ | ✓ | ✓ | ✓ |
| Van d. Heijde 1999^26^ | ASAS | AS | ✓ |  | ✓ | ✓ | ✓ | ✓ | ✓ |  |  | ✓ | ✓ |
| Zochling 2008^27^ | ASAS | AS | ✓ |  |  |  | ✓ | ✓ |  |  | ✓ | ✓ |  |
| Danese 2018^28^ | IOIBD | CD |  |  | ✓ |  |  | ✓ |  |  | ✓ | ✓ |  |
| EMA 2018a^29^ | EMA | CD | ✓ | ✓ | ✓ |  |  |  |  |  |  |  |  |
| Griffiths 2005^30^ | CCFA, NIH | CD |  | ✓ | ✓ |  |  | ✓ |  |  | ✓ | ✓ |  |
| Sahnan 2019^31^ | - | pCD | ✓ |  | ✓ | ✓ |  | ✓ |  |  | ✓ | ✓ | ✓ |
| Sandborn 2002^32^ | IOIBD, CCFA* | CD | ✓ |  | ✓ |  |  |  |  |  | ✓ | ✓ |  |
| Kim 2018^33^ | ICHOM, AFA | IBD | ✓ |  | ✓ | ✓ |  | ✓ |  | ✓ | ✓ | ✓ | ✓ |
| Ruemmele 2015^34^ | PECCO* | IBD |  | ✓ | ✓ |  |  | ✓ |  |  | ✓ | ✓ | ✓ |
| EMA 2015a^35^ | EMA | JIA |  | ✓ | ✓ |  |  |  |  |  |  |  |  |
| Giannini 1997^36^ | ACR* | JIA |  | ✓ | ✓ |  |  |  | ✓ |  | ✓ | ✓ |  |
| EMA 2006^37^ | EMA | PsA | ✓ |  | ✓ |  |  |  |  |  |  |  |  |
| Gladman 2005^38^ | GRAPPA* | PsA | ✓ |  | ✓ | ✓ |  |  |  |  | ✓ | ✓ | ✓ |
| Gladman 2007a^39^ | OMERACT | PsA | ✓ |  | ✓ |  |  |  |  |  | ✓ | ✓ | ? |
| Gladman 2007b^40^ | OMERACT | PsA | ✓ |  | ✓ |  |  |  |  |  |  | ✓ |  |
| Orbai 2017a^41^ | OMERACT | PsA | ✓ |  | ✓ |  |  | ✓ | ✓ | ✓ | ✓ | ✓ | ✓ |
| Orbai 2017b^42^ | OMERACT | PsA | ✓ |  | ✓ |  |  | ✓ | ✓ | ✓ | ✓ | ✓ | ✓ |
| Taylor 2005^43^ | CASPAR | PsA |  |  | ✓ | ✓ | ✓ | ✓ |  |  | ✓ | ✓ |  |
| Tillett 2015^44^ | OMERACT | PsA | ✓ |  | ✓ |  |  |  |  |  | ✓ | ✓ | ✓ |
| Aletaha 2008^45^ | EULAR, ACR | RA | ✓ |  | ✓ |  |  | ✓ |  |  | ✓ | ✓ | ✓ |
| EMA 2018b^46^ | EMA | RA | ✓ |  | ✓ |  |  |  |  |  |  |  |  |
| FDA 2013^47^ | FDA | RA | ✓ |  | ✓ |  |  |  |  |  |  |  |  |
| Felson 1993^48^ | ACR, OMERACT | RA | ✓ |  | ✓ |  |  | ✓ |  |  | ✓ | ✓ |  |
| Felson 1995^49^ | ACR | RA | ✓ |  | ✓ |  |  | ✓ | ✓ |  | ✓ | ✓ |  |
| ICHOM 2018^50^ | ICHOM | RA+ | ✓ | ✓ | ✓ | ✓ |  | ✓ |  |  | ✓ | ✓ | ✓ |
| Kirwan 2007^51^ | OMERACT | RA | ✓ |  | ✓ |  |  | ✓ |  |  | ✓ | ✓ | ✓@ |
| Nikiphorou 2017^52^ | ARUK | RA | ✓ |  | ✓ |  |  |  |  |  | ✓ | ✓ | ✓ |
| Radner 2018^53^ | EULAR | RA | ✓ |  | ✓ | ✓ |  | ✓ | ✓ |  | ✓ | ✓ | ✓ |
| Tugwell 1993^54^ | OMERACT | RA | ✓ |  | ✓ |  |  |  |  |  | ✓ | ✓ |  |
| EMA 2015b^55^ | EMA | SLE | ✓ | ✓ | ✓ |  |  |  |  |  |  |  |  |
| FDA 2010^56^ | FDA | SLE | ✓ | ✓ | ✓ |  |  |  |  |  |  |  |  |
| Gordon 2009^57^ | EULAR | SLE |  |  | ✓ |  |  | ✓ |  |  | ✓ | ✓ |  |
| Liang 2006^58^ | ACR | SLE | ✓ |  | ✓ |  |  | ✓ |  |  | ✓ | ✓ |  |
| Smolen 1999^59^ | OMERACT | SLE | ✓ |  | ✓ |  |  |  |  |  | ✓ | ✓ |  |
| Strand 2000^60^ | OMERACT | SLE | ✓ |  | ✓ |  |  | ✓ |  |  | ✓ | ✓ |  |
| Ruperto 2003^61^ | PRINTO* | jSLE |  | ✓ | ✓ |  |  |  | ✓ |  | ✓ | ✓ |  |
| Ruperto 2006^62^ | PRINTO* | jSLE |  | ✓ | ✓ |  |  |  |  |  | ✓ | ✓ |  |
| Bowman 2001^63^ | - | SS | ✓ |  | ✓ |  |  |  | ✓ |  | ✓ | ✓ |  |
| Pillemer 2005^64^ | - | SS | ✓ |  | ✓ |  |  | ✓ |  |  |  | ✓ |  |
| EMA 2018c^65^ | EMA | UC | ✓ | ✓ | ✓ |  |  |  |  |  |  |  |  |
| FDA 2016^66^ | FDA | UC | ✓ | ✓ | ✓ |  |  |  |  |  |  |  |  |
| Heiligenhaus 2012^67^ | - | Uv |  | ✓ | ✓ |  |  | ✓ | ✓ |  | ✓ | ✓ | X |

EMA – European Medicines Agency

FDA - Food and Drug Administration

ASAS – Assessment of SpondyloArthritis International Society

IOIBD – International Organization for the Study of Inflammatory Bowel Disease

CCFA – Crohn’s and Colitis Foundation of America

AFA – Association Francois Aupetit

NIH – National Institutes of Health

ICHOM - International Consortium for Health Outcomes Measurement

PECCO – Paediatric European Crohn’s and Colitis Organisation (ECCO) committee

ACR – American College of Rheumatology

OMERACT - Outcome Measures in Rheumatology

CASPAR – Classification of Psoriatic Arthritis group

GRAPPA – Group for Research and Assessment of Psoriasis and Psoriatic Arthritis

EULAR - European League Against Rheumatism

ARUK - Arthritis Research UK

PRINTO – Paediatric Rheumatology International Trials Organization

pCD – Fistulising perianal Crohn’s disease

* Multiple affiliations

+ This COS was actually created for ‘inflammatory arthritis’ which includes – RA, PsA, AS and JIA

++ This preliminary set was developed for ‘rheumatic diseases’
