# Supplementary material for: Outcome selection for tissue-agnostic drug trials for immune-mediated inflammatory diseases: a systematic review of core outcome sets and regulatory guidance
Source: Trials. 2022 Jan 15;23:42. doi: 10.1186/s13063-022-06000-w (PMC8761289; doi:10.1186/s13063-022-06000-w)
Supplement: Supplementary file 3 — Additional file 3: Outcome measures proposed across inflammatory conditions [file 13063_2022_6000_MOESM3_ESM.docx]

**Additional file 3**

| Outcome measures proposed across inflammatory conditions | | | | | | | | |
| --- | --- | --- | --- | --- | --- | --- | --- | --- |
| Outcome measures by COS domain | **RA** | **AS** | **PsA** | **SS** | **CD** | **UC** | **Uv** | **SLE** |
| Disease activity | | | | | | | | |
| ACR response criteria (20,50,70) | ✓* |  |  |  |  |  |  |  |
| DAS28 | ✓* |  |  |  |  |  |  |  |
| EULAR response criteria | ✓ |  |  |  |  |  |  |  |
| Clinical activity disease index | ✓ |  |  |  |  |  |  |  |
| CDAI |  |  |  |  | X* |  |  |  |
| SDAI | ✓ |  |  |  |  |  |  |  |
| PDAI |  |  |  |  | ✓ |  |  |  |
| ECLAM-SS |  |  |  | ✓ |  |  |  |  |
| Manitoba IBD Index |  |  |  |  | ✓ | ✓ |  |  |
| PCDAI/wPCDAI |  |  |  |  | ✓P |  |  |  |
| mCDAI |  |  |  |  | ✓P |  |  |  |
| FDA-modified Mayo score |  |  |  |  |  | ✓* |  |  |
| FDA-modified UCDAI score |  |  |  |  |  | ✓* |  |  |
| PUCAI |  |  |  |  |  | ✓P |  |  |
| Slit-lamp examination |  |  |  |  |  |  | ✓ |  |
| Laser flare photometry |  |  |  |  |  |  | ✓ |  |
| SRI |  |  |  |  |  |  |  | ✓* |
| BICLA |  |  |  |  |  |  |  | ✓* |
| ECLAM |  |  |  |  |  |  |  | ✓* |
| SLEDAI |  |  |  |  |  |  |  | ✓* |
| SLAM/SLAM-R |  |  |  |  |  |  |  | ✓* |
| BILAG |  |  |  |  |  |  |  | ✓* |
| LAI |  |  |  |  |  |  |  | ✓ |
| SIS |  |  |  |  |  |  |  | ✓ |
| Flares | | | | | | | | |
| BILAG |  |  |  |  |  |  |  | ✓* |
| SELENA-SLEDAI |  |  |  |  |  |  |  | ✓* |
| Low disease activity | | | | | | | | |
| DAS28–ESR/CRP | ✓* |  |  |  |  |  |  |  |
| SDAI | ✓* |  |  |  |  |  |  |  |
| CDAI | ✓* |  |  |  |  |  |  |  |
| Tender/swollen joints | | | | | | | | |
| ACR response criteria | ✓* |  |  |  |  |  |  |  |
| 28 joint count | ✓ |  |  |  |  |  |  |  |
| 68/66 joint count |  |  | ✓* |  |  |  |  |  |
| 78/76 joint count |  |  | ✓* |  |  |  |  |  |
| BASDAI |  | ✓ |  |  |  |  |  |  |
| PsARC |  |  | ✓* |  |  |  |  |  |
| DAS |  |  | ✓ |  |  |  |  |  |
| EULAR response criteria |  |  | ✓ |  |  |  |  |  |
| CPDAI |  |  | ✓ |  |  |  |  |  |
| PASDAS |  |  | ✓ |  |  |  |  |  |
| GRACE |  |  | ✓ |  |  |  |  |  |
| Enthesitis | | | | | | | | |
| 44-joint count |  | ✓* |  |  |  |  |  |  |
| BASDAI |  | ✓* |  |  |  |  |  |  |
| ASDAS |  | ✓* |  |  |  |  |  |  |
| MASES |  | ✓* | ✓* |  |  |  |  |  |
| Berlin Index |  | ✓ |  |  |  |  |  |  |
| San Francisco (UCSF) Index |  | ✓ |  |  |  |  |  |  |
| CPDAI |  |  | ✓ |  |  |  |  |  |
| PASDAS |  |  | ✓ |  |  |  |  |  |
| SPARCC enthesitis indices |  |  | ✓ |  |  |  |  |  |
| LENIN |  |  | ✓ |  |  |  |  |  |
| Dactylitis | | | | | | | | |
| LDI |  |  | ✓ |  |  |  |  |  |
| CPDAI |  |  | ✓ |  |  |  |  |  |
| PASDAS |  |  | ✓ |  |  |  |  |  |
| Joint/structural damage | | | | | | | | |
| Sharp–van der Heijde score | ✓* |  |  |  |  |  |  |  |
| SASSS score |  | ✓* | ✓* |  |  |  |  |  |
| SPARCC |  | ✓* |  |  |  |  |  |  |
| BASRI |  |  | ✓* |  |  |  |  |  |
| PARS |  |  | ✓* |  |  |  |  |  |
| X-ray | ✓* | ✓ | ✓* |  |  |  |  |  |
| MRI |  | ✓* | ✓ |  |  |  |  |  |
| CT scan |  | ✓* |  |  |  |  |  |  |
| Organ damage | | | | | | | | |
| SDI |  |  |  |  |  |  |  | ✓* |
| Bowel damage progression | | | | | | | | |
| Lémann Index |  |  |  |  | ✓ |  |  |  |
| Patient's global assessment of disease activity | | | | | | | | |
| NRS |  | ✓ | ✓ |  |  |  |  |  |
| VAS |  | ✓* | ✓* |  |  |  |  | ✓P |
| BASDAI |  | ✓* |  |  |  |  |  |  |
| ASAS response criteria |  | ✓* |  |  |  |  |  |  |
| ASDAS |  | ✓* |  |  |  |  |  |  |
| PASDAS |  |  | ✓ |  |  |  |  |  |
| GRACE |  |  | ✓ |  |  |  |  |  |
| Physician global assessment | | | | | | | | |
| VAS |  |  | ✓* |  |  |  |  | ✓ |
| LAI |  |  |  |  |  |  |  | ✓ |
| SLAM |  |  |  |  |  |  |  | ✓ |
| Skin disease activity | | | | | | | | |
| PASI |  |  | ✓ |  |  |  |  |  |
| NPF scoring system |  |  | ✓ |  |  |  |  |  |
| Body surface area |  |  | ✓ |  |  |  |  |  |
| CPDAI |  |  | ✓ |  |  |  |  |  |
| GRACE |  |  | ✓ |  |  |  |  |  |
| Ophthalmic outcome | | | | | | | | |
| Dye score |  |  |  | ✓ |  |  |  |  |
| Schirmers (+/– anaesthetic) |  |  |  | ✓ |  |  |  |  |
| Occular surface damage | | | | | | | | |
| 3 test tool (Schirmer I, Rose  Bengal, lysosome lysis) |  |  |  | ✓ |  |  |  |  |
| Oxford grading scheme |  |  |  | ✓ |  |  |  |  |
| NEI Industry scheme |  |  |  | ✓ |  |  |  |  |
| Global improvement in disease activity | | | | | | | | |
| ASAS response criteria |  | ✓* |  |  |  |  |  |  |
| ASDAS |  | ✓* |  |  |  |  |  |  |
| Clinical remission | | | | | | | | |
| ACR-EULAR | ✓* |  |  |  |  |  |  |  |
| DAS28–ESR/CRP | ✓* |  |  |  |  |  |  |  |
| SDAI | ✓* |  |  |  |  |  |  |  |
| CDAI | ✓* |  |  |  |  |  |  |  |
| FDA-modified Mayo score |  |  |  |  |  | ✓* |  |  |
| FDA-modified UCDAI score |  |  |  |  |  | ✓* |  |  |
| Manitoba IBD Index |  |  |  |  | ✓ |  |  |  |
| PUCAI |  |  |  |  |  | ✓P |  |  |
| Symptomatic remission | | | | | | | | |
| PRO2/PRO3 |  |  |  |  | ✓* |  |  |  |
| Mayo score (clinical section) |  |  |  |  |  | ✓ |  |  |
| PUCAI |  |  |  |  |  | ✓P |  |  |
| Endoscopic remission (Mucosal healing) | | | | | | | | |
| CDEIS |  |  |  |  | ✓* |  |  |  |
| SES-CD |  |  |  |  | ✓* |  |  |  |
| Mayo sub-score (central  readings) |  |  |  |  |  | ✓* |  |  |
| FDA modified Mayo score |  |  |  |  |  | ✓* |  |  |
| Radiological remission | | | | | | | | |
| Bowel wall thickness, dilation,  stricture measurements |  |  |  |  | ✓ |  |  |  |
| Relapse-free corticosteroid-free remission | | | | | | | | |
| PUCAI |  |  |  |  |  | ✓P |  |  |
| Pain | | | | | | | | |
| ACR VAS | ✓* |  |  |  |  |  |  |  |
| VAS (unspec) |  | ✓* | ✓* |  |  |  |  |  |
| NRS | ✓ | ✓* | ✓ |  |  |  |  |  |
| EQ-5D (pain item) | ✓ |  |  |  |  |  |  |  |
| SF-36 (bodily pain) | ✓ | ✓ | ✓ |  |  |  |  |  |
| PROMIS pain interference | ✓ | ✓ | ✓ |  |  |  |  |  |
| BASDAI |  | ✓* |  |  |  |  |  |  |
| IBD-Control |  |  |  |  | ✓ | ✓ |  |  |
| ASDAS | ✓ |  |  |  |  |  |  |  |
| PedsQL - pain | ✓ |  | ✓ |  |  |  |  |  |
| Fatigue | | | | | | | | |
| BRAF | ✓ | ✓ | ✓ |  |  |  |  |  |
| FACIT–F | ✓ | ✓ | ✓ |  |  |  |  | ✓* |
| FSS |  |  |  |  |  |  |  | ✓* |
| BFI |  |  |  |  |  |  |  | ✓* |
| MAF global fatigue index | ✓ |  |  |  |  |  |  |  |
| SF-36 (vitality) | ✓ |  |  | ✓ |  |  |  |  |
| BASDAI (Fatigue item) |  | ✓* |  |  |  |  |  |  |
| IBD-Control questionnaire |  |  |  |  | ✓ | ✓ |  |  |
| NRS | ✓ | ✓ | ✓ |  |  |  |  |  |
| VAS | ✓ | ✓ | ✓ | ✓ |  |  |  |  |
| PROMIS-fatigue | ✓ | ✓ | ✓ |  |  |  |  |  |
| PedsQL 4.0 (fatigue total score) |  | ✓ | ✓ |  |  |  |  |  |
| PROFAD |  |  |  | ✓ |  |  |  |  |
| Sicca symptoms | | | | | | | | |
| OSDI |  |  |  | ✓ |  |  |  |  |
| SSI |  |  |  | ✓ |  |  |  |  |
| Bowel symptoms | | | | | | | | |
| IBD-Control questionnaire |  |  |  |  | ✓ | ✓ |  |  |
| Xerostomia inventory |  |  |  | ✓ |  |  |  |  |
| FDA-modified Mayo score |  |  |  |  |  | ✓* |  |  |
| FDA-modified UCDAI score |  |  |  |  |  | ✓* |  |  |
| Function/Disability | | | | | | | | |
| ADL |  |  |  |  |  |  |  | ✓* |
| ASAS |  | ✓* |  |  |  |  |  |  |
| HAQ | ✓ |  | ✓* |  |  |  |  |  |
| HAQ-II | ✓ |  | ✓ |  |  |  |  |  |
| HAQ-DI | ✓* |  | ✓ |  |  |  |  |  |
| HAQ-S |  |  | ✓* |  |  |  |  |  |
| HAQSK |  |  | ✓* |  |  |  |  |  |
| MD-HAQ | ✓ |  | ✓ |  |  |  |  |  |
| MHAQ | ✓ |  |  |  |  |  |  |  |
| C-HAQ | ✓ |  | ✓ |  |  |  |  |  |
| EQ-5D – function item | ✓ |  |  |  |  |  |  |  |
| BASFI | ✓ | ✓* | ✓ |  |  |  |  |  |
| AIMS | ✓ |  |  |  |  |  |  |  |
| QWB | ✓ |  |  |  |  |  |  |  |
| MHIQ | ✓ |  |  |  |  |  |  |  |
| MACTAR | ✓ |  |  |  |  |  |  |  |
| JAMAR | ✓ |  |  |  |  |  |  |  |
| PROMIS – physical function | ✓ |  | ✓ |  |  |  |  |  |
| CPDAI |  |  | ✓ |  |  |  |  |  |
| GRACE |  |  | ✓ |  |  |  |  |  |
| VAS |  |  |  |  |  |  | ✓P |  |
| HRQOL | | | | | | | | |
| ASQoL |  | ✓* |  |  |  |  |  |  |
| ASAS Health Index |  | ✓* |  |  |  |  |  |  |
| EQ-5D | ✓ | ✓* | ✓ |  |  |  |  |  |
| SF-6D | ✓ | ✓ | ✓ |  |  |  |  |  |
| SF-36 |  | ✓* | ✓* |  |  |  |  | ✓* |
| Lupus/SLE QoL |  |  |  |  |  |  |  | ✓* |
| PsAQoL |  |  | ✓* |  |  |  |  |  |
| DLQI |  |  | ✓ |  |  |  |  |  |
| CPDAI |  |  | ✓ |  |  |  |  |  |
| PASDAS |  |  | ✓ |  |  |  |  |  |
| GRACE |  |  | ✓ |  |  |  |  |  |
| NRS (Patient Global Assessment) | ✓ | ✓ | ✓ |  |  |  |  |  |
| VAS (Patient Global Assessment) | ✓ | ✓ | ✓ |  |  |  |  |  |
| PROMIS – Global Health | ✓ | ✓ |  |  |  |  |  |  |
| RAID | ✓ | ✓ | ✓ |  |  |  |  |  |
| PSAID |  | ✓ | ✓ |  |  |  |  |  |
| PedsQL |  | ✓P |  |  |  |  | ✓P |  |
| PROMIS – Global Health  Paediatric |  | ✓ |  |  |  |  |  |  |
| EQ-5D-Y |  | ✓ |  |  |  |  |  |  |
| WPAI-GH |  | ✓* |  |  |  |  |  |  |
| JAMAR |  |  | ✓ |  |  |  |  |  |
| CIHQ |  |  | ✓ |  |  |  |  |  |
| NEI-VFQ |  |  |  | ✓ |  |  |  |  |
| OSDI |  |  |  | ✓ |  |  |  |  |
| IBDQ |  |  |  |  | ✓* | ✓* |  |  |
| IMPACT III |  |  |  |  | ✓P | ✓P |  |  |
| CHAQ |  |  |  |  |  |  | ✓P |  |
| Child Health Questionnaire |  |  |  |  |  |  | ✓P | ✓*P |
| Utility | | | | | | | | |
| EQ-5D | ✓ |  |  |  |  |  |  |  |
| Spinal mobility | | | | | | | | |
| Modified Schober |  | ✓* |  |  |  |  |  |  |
| Chest expansion |  | ✓* |  |  |  |  |  |  |
| Occiput to wall |  | ✓* |  |  |  |  |  |  |
| Cervical rotation |  | ✓* |  |  |  |  |  |  |
| Lateral spine flexion |  | ✓* |  |  |  |  |  |  |
| BASMI |  | ✓* |  |  |  |  |  |  |
| Spinal stiffness | | | | | | | | |
| ASDAS |  | ✓* |  |  |  |  |  |  |
| ASAS response criteria |  | ✓* | ✓* |  |  |  |  |  |
| BASDAI |  | ✓* | ✓* |  |  |  |  |  |
| CPDAI |  |  | ✓ |  |  |  |  |  |
| NRS |  | ✓* |  |  |  |  |  |  |
| VAS |  | ✓* |  |  |  |  |  |  |
| Nutritional status | | | | | | | | |
| BMI |  |  |  |  | ✓ | ✓ |  |  |
| Work limitation/productivity | | | | | | | | |
| WPAI | ✓ | ✓ | ✓ |  |  |  |  | ✓* |
| PedsQL 4.0 – School function |  | ✓ | ✓ |  |  |  |  |  |
| Social outcome | | | | | | | | |
| Absence from school |  |  |  |  |  |  | ✓P |  |
| Biomarkers | | | | | | | | |
| Rheumatoid factor | ✓ |  |  |  |  |  |  |  |
| Anti-CCP antibodies | ✓ |  |  |  |  |  |  |  |
| Anti-DNA |  |  |  |  |  |  |  | ✓P |
| ESR | ✓* | ✓* | ✓* |  | ✓ | ✓ |  |  |
| CRP | ✓* | ✓* | ✓* |  | ✓* | ✓* |  |  |
| IgG |  |  |  | ✓ |  |  |  |  |
| Faecal calprotectin |  |  |  |  | ✓* | ✓* |  |  |
| Lactoferrin |  |  |  |  |  | ✓* |  |  |
| Laboratory indices | | | | | | | | |
| 24-hour proteinuria |  |  |  |  |  |  |  | ✓*P |
| Serum creatinine |  |  |  |  |  |  |  | ✓P |
| Spot urine protein: creatinine  ratio |  |  |  |  |  |  |  | ✓*P |
| SAE/Toxicity | | | | | | | | |
| Renal function | ✓* |  |  |  |  |  |  |  |
| Vital signs (e.g. BP) | ✓* |  |  |  |  |  |  |  |
| Immune system function |  | ✓* |  |  |  |  |  |  |
| CTCAE |  |  |  |  |  |  |  | ✓ |
| Overall survival | | | | | | | | |
| Date of death |  |  |  |  | ✓ | ✓ |  |  |
| Long-term efficacy | | | | | | | | |
| Time to relapse |  |  |  |  | ✓ |  |  |  |
| CD-related surgery |  |  |  |  | ✓ |  |  |  |
| Growth | | | | | | | | |
| Height velocity (z score) |  |  |  |  | ✓*P | ✓P |  |  |
| Weight and height |  |  |  |  |  | ✓*P |  | ✓P |
| PCDAI |  |  |  |  | ✓P |  |  |  |
| Tanner puberty stage |  |  |  |  |  |  |  | ✓P |
| Menses |  |  |  |  |  |  |  | ✓P |

* suggested by either FDA or EMA

X – measure not recommended

P – measure proposed for paediatric trials

QWB - Quality of Well Being; MASES - Maastricht AS Enthesitis Score ; SDAI - Simplified disease activity index; wPCDAI - weighted Paediatric Crohn’s Disease Activity Index; CHAQ - Childhood Health Assessment Questionnaire; CDEIS – Crohn’s Disease Endoscopic Index of Severity; SES-CD - Simple Endoscopic Score for Crohn’s Disease; PUCAI - Paediatric Ulcerative Colitis Activity Index; CRP - C reactive protein;

DLQI - Dermatology Life Quality Index; NPF - National Psoriasis Foundation scoring system;

SPARCC - Spondyloarthritis Research Consortium Canada; LENIN - Leeds enthesitis index; PsARC - Psoriatic Arthritis Response Criteria; LDI - Leeds Dactylitis Index; DAS - Disease Activity Score; HAQ - Health Assessment Questionnaire; SF-36 - Medical Outcomes Study Short Form-36; PASI - Psoriatic Activity and Severity Index; GRACE - GRAppa Composite Exercise; CPDAI - Composite Psoriatic Disease Activity Index;

PASDAS - Psoriatic Arthritis Disease Activity Score; BASDAI - Bath Ankylosing Spondylitis Disease Activity Index; ACR - American College of Rheumatology; PARS - Psoriatic Arthritis Ratingen Score; BASRI - Bath Ankylosing Spondylitis Radiology Index; SASSS - Stoke Ankylosing Spondylitis Spine Score;

mSASSS - Modified Stoke Ankylosing Spondylitis Spine Score; ESR - Erythrocyte sedimentation rate;

WPAI - Work Productivity and Activity Impairment Questionnaire; SRI - SLE Responder Index; BILAG - British Isles Lupus Assessment Group; BICLA - BILAG-based Composite Lupus Assessment;

ECLAM - European Consensus Lupus Activity Measure; SLEDAI - Systemic Lupus Erythematosus Disease Activity Index; SLAM - Systemic Lupus Erythematosus Activity Measure; LAI - Lupus Activity Index;

SIS - National Institutes of Health SLE Index Score; SLICC Systemic Lupus Erythematosus International Collaborating Clinics; SDI - SLICC/ACR Damage Index
